# Development of a rapid and highly accurate method for ^13^C tracer-based metabolomics and its application on a hydrogenotrophic methanogen

**DOI:** 10.1093/ismeco/ycad006

**Published:** 2024-01-10

**Authors:** Yuto Fukuyama, Shigeru Shimamura, Sanae Sakai, Yuta Michimori, Tomomi Sumida, Yoshito Chikaraishi, Haruyuki Atomi, Takuro Nunoura

**Affiliations:** Research Center for Bioscience and Nanoscience (CeBN), Japan Agency for Marine-Earth Science and Technology (JAMSTEC), 2–15 Natsushima-cho, Yokosuka, Kanagawa 237–0061, Japan; Institute for Extra-Cutting-Edge Science and Technology Avant-Garde Research (X-star), Japan Agency for Marine-Earth Science and Technology (JAMSTEC), 2–15 Natsushima-cho, Yokosuka, Kanagawa 237–0061, Japan; Institute for Extra-Cutting-Edge Science and Technology Avant-Garde Research (X-star), Japan Agency for Marine-Earth Science and Technology (JAMSTEC), 2–15 Natsushima-cho, Yokosuka, Kanagawa 237–0061, Japan; Department of Synthetic Chemistry and Biological Chemistry, Graduate School of Engineering, Kyoto University, Katsura, Nishikyo-ku, Kyoto 615-8510, Japan; Research Center for Bioscience and Nanoscience (CeBN), Japan Agency for Marine-Earth Science and Technology (JAMSTEC), 2–15 Natsushima-cho, Yokosuka, Kanagawa 237–0061, Japan; Institute of Low Temperature Science, Hokkaido University, Kita-19, Nishi-8, Kita-ku, Sapporo 060-0819, Japan; Department of Synthetic Chemistry and Biological Chemistry, Graduate School of Engineering, Kyoto University, Katsura, Nishikyo-ku, Kyoto 615-8510, Japan; Research Center for Bioscience and Nanoscience (CeBN), Japan Agency for Marine-Earth Science and Technology (JAMSTEC), 2–15 Natsushima-cho, Yokosuka, Kanagawa 237–0061, Japan

**Keywords:** capillary electrophoresis-tandem mass spectrometry (CE-MS), ^13^C tracer, metabolomics, methanogen

## Abstract

Microfluidic capillary electrophoresis-mass spectrometry (CE-MS) is a rapid and highly accurate method to determine isotopomer patterns in isotopically labeled compounds. Here, we developed a novel method for tracer-based metabolomics using CE-MS for underivatized proteinogenic amino acids. The method consisting of a ZipChip CE system and a high-resolution Orbitrap Fusion Tribrid mass spectrometer allows us to obtain highly accurate data from 1 μl of 100 nmol/l amino acids comparable to a mere 1 $\times$ 10^4^–10^5^ prokaryotic cells. To validate the capability of the CE-MS method, we analyzed 16 protein-derived amino acids from a methanogenic archaeon *Methanothermobacter thermautotrophicus* as a model organism, and the mass spectra showed sharp peaks with low mass errors and background noise. Tracer-based metabolome analysis was then performed to identify the central carbon metabolism in *M. thermautotrophicus* using ^13^C-labeled substrates. The mass isotopomer distributions of serine, aspartate, and glutamate revealed the occurrence of both the Wood–Ljungdahl pathway and an incomplete reductive tricarboxylic acid cycle for carbon fixation. In addition, biosynthesis pathways of 15 amino acids were constructed based on the mass isotopomer distributions of the detected protein-derived amino acids, genomic information, and public databases. Among them, the presence of alternative enzymes of alanine dehydrogenase, ornithine cyclodeaminase, and homoserine kinase was suggested in the biosynthesis pathways of alanine, proline, and threonine, respectively. To our knowledge, the novel ^13^C tracer-based metabolomics using CE-MS can be considered the most efficient method to identify central carbon metabolism and amino acid biosynthesis pathways and is applicable to any kind of isolated microbe.

## Introduction

Metabolomics is an effective method to detect the metabolic states of microbial cells. Intracellular metabolite levels are regulated in a complex manner responding to metabolite generation and/or consumption brought about by enzymatic, nonenzymatic, and transport reactions [1, 2]. For over the past two decades, ^13^C tracer analysis has commonly been utilized as an accurate and physiologically reliable method to examine the changes in intracellular metabolite levels (metabolic fluxes) [3]. ^13^C tracer-based metabolomics provide the means to identify metabolic pathways and their direction because the patterns of the detected ^13^C-labeled metabolites reflect the amount of metabolic flux [1]. Based on ^13^C labeling patterns in key metabolites, active pathways can be traced back, and in some cases the presence of new enzymes can be revealed. In addition, tracking metabolic flux under different growth phases and/or conditions should unveil variations of metabolic functions in certain microbes.

To date, several approaches to identify and quantify intermediate metabolites have been examined using gas chromatography (GC) with mass spectrometry (GC–MS), liquid chromatography (LC) with mass spectrometry (LC–MS), or LC with nuclear magnetic resonance [4-6]. Currently, GC–MS, which is more sensitive than the other methods, is the most popular technique to determine isotopologues of amino acids and subsequently identify novel carbon fixation pathways [7, 8]. However, even with the high sensitivity of the GC–MS technique, large biomass grown with ^13^C-labeled substrates is sometimes required [9]. In addition, background noises of the mass spectra from GS–MS data interfere with the identification of ^13^C-labeled compounds and the determination of ^13^C distribution. Thus, to overcome these issues, analytical software to interpret GC–MS data has been developed for the isotopomer analysis of ^13^C-labeled amino acids [10]. For instance, MassWorks software and Isotopo software, a formula determination tool, provide spectra accuracy based on exact calibration from relatively small biomass samples [11-14]. From another point of view, because volatilization of the analytes is required for GC–MS analysis, derivatization of each chemical moiety is necessary for analyzing nonvolatile metabolites to improve sensitivity and separation in the chromatography. However, difficulties in data interpretation often occur in ^13^C tracer-based metabolomics using GC–MS because many carbons deriving from the derivative groups are incorporated. In addition, multiple-step derivatization requires time-consuming and complicated procedures. Thus, the development of a highly accurate and simple analytical method is one of the most demanding challenges in tracer-based metabolomics.

Recently, capillary electrophoresis coupled to mass spectrometry (CE-MS) has been developed as a simple technique for metabolomic studies. The CE-MS method does not require abundant biomass nor derivatization of molecules for analysis and provides data with low background noise [15, 16]. Other advantages of CE-MS include high separation efficiencies, tiny sample injection volumes (nL range), and low reagent costs [17]. The method can thus be expected to be applicable for tracer-based metabolomics to obtain high-quality data from less biomass compared to GC–MS-based metabolomics. However, the limitation is that CE often lacks sensitivity due to low sample loading capacity when coupled with a general detector [18], and thus, CE has not been a popular tool in metabolomics.

*Methanothermobacter thermautotrophicus* is a model organism of thermophilic and hydrogenotrophic methanogens. The methanogen grows chemolithoautotrophically by generating methane using H_2_ and CO_2_ as sole energy and carbon sources, respectively [19-21]. In this methanogen, CO_2_ is sequentially reduced to methane via the archaeal type Wood–Ljungdahl (WL) pathway for energy conservation and to acetyl-CoA for carbon fixation. In addition, in combination with classic tracer-based metabolomics and genomic information, the capability of *M. thermautotrophicus* to fix carbon via an incomplete reductive tricarboxylic acid (rTCA) cycle has also been proposed [22-25]. From genomic information, the organism harbors most enzymes of the TCA cycle but lacks aconitase hydratase (EC 4.2.1.3) and isocitrate dehydrogenase (EC 1.1.1.42) [24]. The metabolic function of the putative incomplete TCA cycle has been partly revealed by several radioisotope tracer analyses using ^14^C-labeled substrates, including acetate [22, 23]. The methanogen cannot utilize acetate as a substrate for methanogenesis under physiological conditions [19]. However, acetate is assimilated as a carbon source via acetyl-CoA carboxylation to pyruvate and subsequent incorporation into glutamate (Glu) via reductive reactions of the TCA cycle [23, 26]. However, experimental evidence is limited to verify the metabolic function of central carbon metabolism in *M. thermautotrophicus* and the roles of the incomplete TCA cycle.

Here, we developed a novel, rapid, sensitive, and highly accurate tracer-based metabolomics method for proteinogenic amino acids using CE-MS and CE-MS/MS. Combining the recently developed ZipChip CE system and the Orbitrap Fusion Tribrid mass spectrometer overcomes the limitation of previously established CE-MS systems. The ZipChip CE system is a microfluidic CE device providing quick separation and sensitive detection owing to nano-electrospray ionization. The Orbitrap Fusion Tribrid mass spectrometer provides ultra-high resolution for improved structural elucidation and determination of isotopomer patterns in isotopically labeled compounds. Thus, the novel method enables us to trace metabolic pathways from underivatized and tiny amounts of analytes. Specifically, the analytical method requires only 1 μl of 100 nmol/l amino acids, which corresponds to those from ~10^4^ to 10^5^ prokaryotic cells. Total run time, including washing steps, is 15 min for each sample. Moreover, the method reveals the position of labeled carbon using CE-MS/MS analysis, which is essential to understand the carbon metabolic pathways and amino acid biosynthesis pathways. In this study, the contribution of the incomplete rTCA cycle in *M. thermautotrophicus* metabolism was examined based on the isotopologues and isotopomer analysis as the first ^13^C tracer-based metabolomics report using the combination of the ZipChip CE system and the Orbitrap Fusion Tribrid mass spectrometer. Furthermore, all amino acid biosynthesis pathways were reconstructed. A dataset of the ^13^C tracer-based metabolomics together with genomic information provides the most probable central carbon metabolism in this methanogen along with its amino acid biosynthesis pathways. The novel CE-MS and CE-MS/MS techniques are promising tools to reveal the amino acid biosynthesis pathways and related central carbon metabolism that are essential to understand the core anabolism of life in phylogenetically and metabolically diverse organisms, including those that are not applicable for metabolomics because of the difficulties to obtain sufficient biomass.

## Materials and methods

### Strain and growth conditions

*M. thermautotrophicus* delta H^T^ (=JCM10044^T^) was obtained from the Japan Collection of Microorganisms (JCM) in Riken. Unless indicated otherwise, *M. thermautotrophicus* was grown in 3 ml of JCM231 medium in 17 ml test tubes at 65°C. Headspace gas was balanced to atmospheric pressure with H_2_/CO_2_ [80:20, (v/v)].

#### *In vivo incorporation* of ^13^C-labeled substrates

All the ^13^C-labeled reagents used in this study were purchased from Cambridge isotope laboratories (Tewksbury, MA). For tracer-based metabolomics, *M. thermautotrophicus* was grown in the JCM231 medium with ^13^C-labeled substrate. After 24 h of cultivation, 0.14 ml ^13^CO_2_ was added to the headspace of the culture [final concentration, 5% (v/v) of the headspace CO_2_]. [1-^13^C_1_] or [2-^13^C_1_] sodium acetate was added in JCM231 medium [final concentration, 0.01% (wt/v)] prior to inoculation. Cells in the 3 ml-culture each with ^13^C-labeled reagent were harvested at the exponential phase (48 h after incubation) by centrifugation (4°C, 10000 $\times$  *g*, 10 min), frozen in liquid N_2_, and then stored at −80°C until use. Negative control cultures were also prepared under the same conditions without ^13^C-labeled substrates. All isotope tracing experiments were performed in duplicate.

### Sample preparation

To prepare protein-derived amino acids, the stored cells were hydrolyzed in 12 N HCl at 110°C for 16 h in a 1.0 ml glass reaction vial (GL Science, Tokyo, Japan). After adding *n*-hexane to remove hydrophobic constituents, the hydrolysate was filtered through Nanosep MF centrifugal devices with 0.45 μm wwPTFE membrane (PALL, Port Washington, NY) by centrifugation. The filtered sample was washed with *n*-hexane/dichloromethane [1:2, (v/v)] three times. The dried protein-derived amino acids were obtained with evaporation under an N_2_ stream and then stored at −80°C until use.

### Isotopologue and isotopomer analyses

Isotope abundance and incorporation rate were examined using a ZipChip CE system coupled with an Orbitrap Fusion Tribrid mass spectrometer (Thermo Fisher Scientific, Waltham, MA). The CE separation was performed with an HS chip (908 Devices, Boston, MA) in the following manner: injection volume 5 nL, field strength 1000 V/cm, analysis run time 3 min without pressure assist. The MS analysis was operated in positive ion mode to detect targeted proteinogenic amino acids based on their accurate masses. The full scan MS settings included: Detector Orbitrap, Resolution 15 000, Quadrupole Isolation ON, Scan Range 70–500 *m/z*, an acquisition gain control (AGC) Target Standard, Maximum Injection Time Mode 50 ms, EASY-IC ON, Sheath Gas Flow Rate 2.2 arbitrary units, Capillary Temperature at 200°C, S-lens Radio Frequency Level 60%. The MS/MS settings included: Isolation Mode quadrupole, Isolation Window 0.7 *m/z*, Activation Type HCD, Collision Energy Mode stepped (25, 35, 50), Detector Type Orbitrap, Resolution 15 000, Scan Range Mode Define First Mass (*m/z* 40), AGC Target Standard, Maximum Injection Time Mode Dynamic. The obtained data were analyzed using Qual Browser in Xcalibur version 4.3.73.11 (Thermo Fisher Scientific). Prior to the analysis for the protein-derived amino acids, a complete standard mixture of amino acids (Promega, Medison, WI) was analyzed by CE-MS to obtain MS and MS/MS spectra of the proteinogenic amino acids. Nonlabeled, [1-^13^C_1_], [5-^13^C_1_], and [^13^C_5_] Glu were also examined for validation of the capability of MS/MS fragmentation. Detected protein-derived amino acids were identified by migration times and monoisotopic mass matching to the amino acid mixture under the same condition. ^13^C-labeled positions and their ^13^C abundance were estimated by fragment patterns based on a ^13^C-labeled standard of amino acids and an MS spectra library (mzCloud) (Thermo Fisher Scientific).

To identify the central carbon metabolism and amino acid biosynthesis pathways based on the isotopologue patterns of amino acids, we compared the observed mass isotopomer distributions with predicted labeling structures from genomic information in Kyoto Encyclopedia of Genes and Genomes (KEGG) PATHWAY database [27] and MetaCyc [28]. Manual curation of amino acid biosynthesis pathways was performed by a web tool, GapMind [29].

## Results and discussion

### Identification of amino acids using the capillary electrophoresis-mass spectrometry method

**Figure 1:**
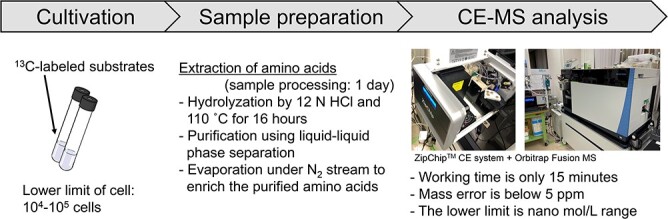
Analytical workflow of ^13^C tracer-based metabolomics using the developed CE-MS.

**Figure 2:**
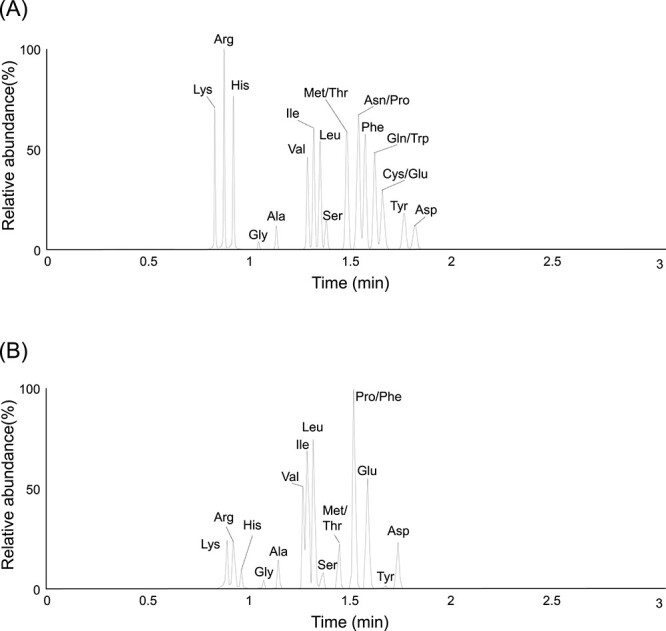
Mass spectra of the detected amino acids from the standard mixture of amino acids (A) and *M. thermautotrophicus* (B); Lys lysine, Arg arginine, Val valine, Leu leucine, Ile isoleucine, Tyr tyrosine, Phe phenylalanine, Met methionine.

To examine the capability of the CE-MS method for identification of amino acids, we analyzed a standard mixture of amino acids (Fig. 1). All amino acids are positively charged in this method using an acidic running buffer and are thus separated in response to the differences in electrophoretic mobility based on each side chain structure. Accordingly, positively charged amino acids were identified in shorter migration times followed by neutral amino acids and then the acidic amino acids, with good reproducibility (Fig. 2A). We next analyzed 20 protein-derived amino acids from *M. thermautotrophicus* cells grown with nonlabeled substrate (Fig. 2B). Sharp peaks of 16 proteinogenic amino acids were identified with slight background noise in a short run (3 min), while those of cysteine (Cys), tryptophan (Trp), asparagine (Asn), and glutamine (Gln) were not detected. It has been pointed out that Cys and Trp are degraded during hydrolyzation with 12 N HCl at high temperature [9, 30]. In addition, Asn and Gln are converted to aspartate (Asp) and Glu, respectively, in 12 N HCl [9, 30]. The mass spectra of the detected cellular protein-derived amino acids were consistent with those from the standard amino acid mixture. Mass errors ranged from 0.04 to 4.61 ppm when we compared the theoretical *m/z* and an experimentally observed *m/z*. Peak areas of the detected amino acids ranged from 4119 to 20 448 890. The lower limit of this method was 1 μl of 100 nmol/l mol amino acids that correspond to the levels of amino acids from ~10^4^ to 10^5^ prokaryotic cells. These results indicated high accuracy and sensitivity of the developed CE-MS method.

### Isotopologue characterization using the capillary electrophoresis-mass spectrometry /mass spectrometry method

**Figure 3:**
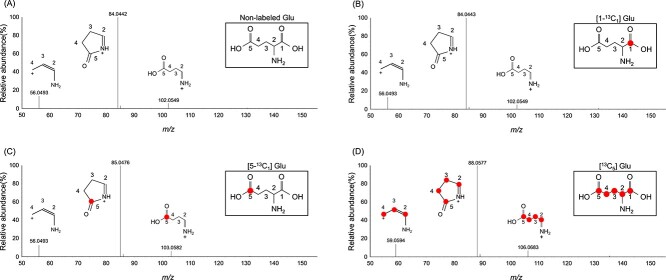
MS/MS spectra of nonlabeled Glu (A), [1-^13^C_1_] Glu (B), [5-^13^C_1_] Glu (C), and [^13^C_5_] Glu (D); Circles show ^13^C in the carbon skeleton.

To validate the applicability of the developed CE-MS/MS method for isotopomer analysis, we analyzed MS and MS/MS spectra of nonlabeled, [1-^13^C_1_], [5-^13^C_1_], and [^13^C_5_] Glu (Fig. 3). The observed labeled fragments in positive ionization were consistent with the numbers and localization of the labeled carbons in each Glu. In conventional tracer-based metabolomics using GC–MS, high-accuracy data are adjusted by analytical software to distinguish ^13^C distribution in each derivatized amino acid. By contrast, the developed CE/MS and CE-MS/MS methods provided sufficiently clear and accurate MS/MS spectra without complicated procedures of multiple-step derivatization for sample pretreatment. The method also provides an advantage in practical working time. Although GC–MS analysis took 30–60 min for derivatization, 10–20 min for separation, and 10 min for equilibration per sample, the developed method took only 15 min, including washing time. Thus, we concluded that the developed method was applicable in providing practical clues to characterize isotopologues of amino acids.

### Occurrence of both the Wood–Ljungdahl pathway and an incomplete reductive tricarboxylic acid cycle in *M. thermautotrophicus*

**Figure 4:**
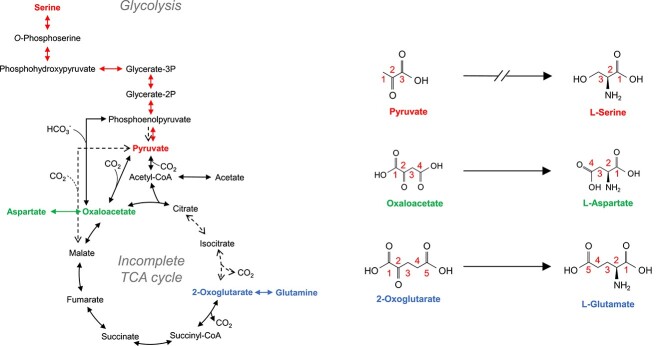
Carbon transitions of Ser, Asp, and Glu via each biosynthesis pathway based on genomic information; numbers in each structural formula show carbon numbers.

To verify the central carbon metabolism and amino acid biosynthesis pathways in *M. thermautotrophicus*, we applied the CE-MS/MS method for isotopomer analysis. The methanogen is expected to harbor the WL pathway and an incomplete rTCA cycle for carbon fixation based on genomic analyses [24, 25]. Genomic information suggests that serine (Ser), Asp, and Glu are synthesized from pyruvate, oxaloacetate, and 2-oxoglutarate, respectively (Fig. 4). In this study, ^13^CO_2_ [final concentration, 5% (v/v) of the headspace CO_2_] and ^13^C labeled acetate [final concentration, 0.01% (wt/v)] were used as a tracer to identify the carbon fixation route of the methanogen. The low concentration of the ^13^C labeled acetate was expected not to affect the metabolic flux of the inorganic carbon fixation pathways.

**Figure 5:**
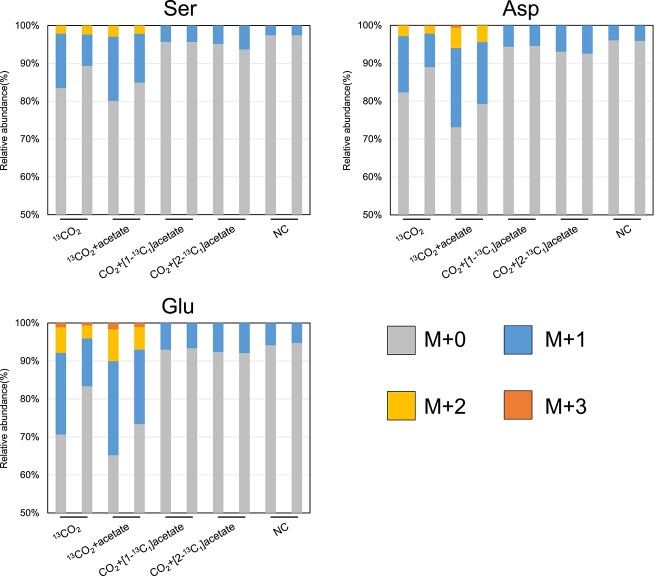
Mass isotopomer distributions from selected amino acids for the WL pathway, the incomplete TCA cycle, and gluconeogenesis; the mass fractions for M + 0, M + 1, M + 2, and M + 3 represent fragments containing 0–3 ^13^C-labeled carbons, respectively.

**Figure 6:**
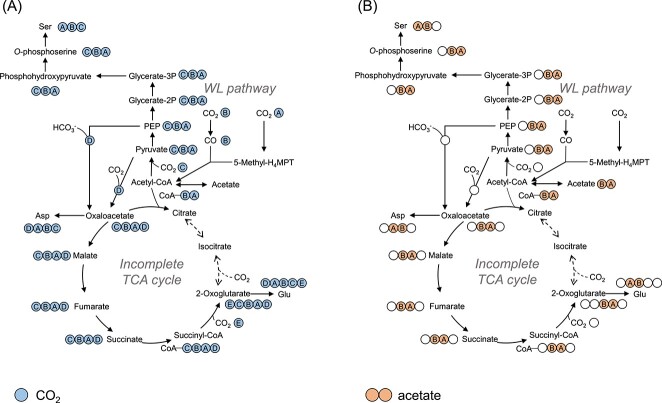
Elucidating the occurrence of both the WL pathway and the incomplete rTCA cycle; proposed labeling of metabolites from metabolome analysis with ^13^CO_2_ (A) and ^13^C-labeled acetate ([1-^13^C_1_] or [2-^13^C_1_] acetate) (B) during the WL pathway and incomplete TCA cycle; circles indicate carbon atoms in the metabolites; dotted lines indicate that there are no corresponding enzymes found in the genome.

In cells grown with nonlabeled substrates, isotopomers labeled with natural ^13^C were detected in the mass fractions of M + 1 (Fig. 5, Table 1). The relative abundance of the mass fraction labeled with natural ^13^CO_2_ (M + 1 fraction) in Ser, Asp, and Glu varied from each other. The result suggested that the incorporation of natural ^13^CO_2_ affected the isotopomer composition of the precursors of Ser, Asp, and Glu. Thus, the incorporation rate of ^13^C *in vivo* labeling was evaluated after eliminating the natural abundance of ^13^C (1.1%) (Table 1). In cells grown with added ^13^CO_2_ (final concentration, 5% v/v in the headspace CO_2_), up to three labeled carbons were detected in Ser (Fig. 5 and Table 1), and ^13^C incorporation was found in both main- and side-chains (Table 1). The isotopomer patterns showed their autotrophic growth using CO_2_ as the sole carbon source. From their genomic information, the methanogen was expected to fix carbon via the WL pathway [24], followed by carboxylation of acetyl-CoA to form pyruvate and by Ser biosynthesis from pyruvate via 3-phosphoglycerate. Similarly, Asp harbored up to three labeled carbons (Fig. 5 and Table 1). The ^13^C incorporation in the side-chain of Asp is explained by the carboxylation of pyruvate to form oxaloacetate (the precursor of Asp), and subsequent Asp biosynthesis from oxaloacetate (Fig. 5A). Glu also had up to three labeled carbons (Fig. 5 and Table 1). The ^13^C incorporation at the main-chain of Glu can be explained by the carboxylation of succinyl-CoA to form 2-oxoglutarate (Fig. 6A), and subsequent Glu biosynthesis from 2-oxoglutarate. If a complete rTCA cycle occurred, acetyl-CoA (the precursor of pyruvate) and oxaloacetate (the precursor of Asp) should be derived from citrate that is synthesized from 2-oxoglutarate (the precursor of Glu) via reductive reactions. However, an abundance of the labeled carbon in Ser and Asp was lower than that of Glu (Fig. 5). The result suggested that the rTCA cycle was incomplete.

**Table 1:** The mass isotopomer distributions of Ser, Asp, and Glu from the developed CE-MS analysis.

	Natural abundance of isotopes	^13^CO_2_		^13^CO_2_ + acetate		CO_2_ + [1-^13^C_1_]acetate		CO_2_ + [2-^13^C_1_]acetate		Negative control	
		Replicate 1	Replicate 2	Replicate 1	Replicate 2	Replicate 1	Replicate 2	Replicate 1	Replicate 2	Replicate 1	Replicate 2
Amino acid	Abundance (%)	Abundance (%)	Abundance (%)	Abundance (%)	Abundance (%)	Abundance (%)	Abundance (%)	Abundance (%)	Abundance (%)	Abundance (%)	Abundance (%)
^13^C_0_ Ser	96.74	84.99	92.89	80.51	86.59	95.73	96.23	95.19	94.04	96.89	96.86
^13^C_1_ Ser	3.23	13.45	6.38	17.62	11.30	4.27	3.77	4.81	5.96	3.11	3.14
^13^C_2_ Ser	0.04	1.57	0.74	1.87	2.11	N.D.	N.D.	N.D.	N.D.	N.D.	N.D.
^13^C_3_ Ser	0.00	N.D.	N.D.	0.12	0.04	N.D.	N.D.	N.D.	N.D.	N.D.	N.D.
^13^C_0_ Asp	95.67	78.93	89.01	71.48	80.21	95.26	94.66	93.77	92.55	95.88	96.28
^13^C_1_ Asp	4.26	16.36	8.30	21.42	15.16	4.74	5.34	6.23	7.45	4.12	3.72
^13^C_2_ Asp	0.07	4.31	2.42	6.34	4.26	N.D.	N.D.	N.D.	N.D.	N.D.	N.D.
^13^C_3_ Asp	0.00	0.40	0.27	0.76	0.37	N.D.	N.D.	N.D.	N.D.	N.D.	N.D.
^13^C_4_ Asp	0.00	N.D.	N.D.	N.D.	N.D.	N.D.	N.D.	N.D.	N.D.	N.D.	N.D.
^13^C_0_ Glu	94.62	71.81	84.11	65.74	73.99	93.05	93.52	92.35	91.52	94.67	94.54
^13^C_1_ Glu	5.26	20.49	11.88	23.97	18.83	6.95	6.48	7.65	8.48	5.33	5.46
^13^C_2_ Glu	0.12	6.51	3.14	8.54	6.07	N.D.	N.D.	N.D.	N.D.	N.D.	N.D.
^13^C_3_ Glu	0.00	1.19	0.88	1.75	1.11	N.D.	N.D.	N.D.	N.D.	N.D.	N.D.
^13^C_4_ Glu	0.00	N.D.	N.D.	N.D.	N.D.	N.D.	N.D.	N.D.	N.D.	N.D.	N.D.
^13^C_5_ Glu	0.00	N.D.	N.D.	N.D.	N.D.	N.D.	N.D.	N.D.	N.D.	N.D.	N.D.

(*N.D., not detected*)

To confirm the operation of an incomplete rTCA cycle, we also examined the isotopomer analysis using ^13^C labeled acetate: [1-^13^C_1_] or [2-^13^C_1_] acetate [final concentration, 0.01% (wt/v)]. The ^13^C incorporation pattern showed that the relative abundance of mass fraction of M + 1 in each amino acid increased compared to those in the negative control (Fig. 5 and Tables 1 and 2). In addition, the labeled carbon was also observed in the side-chain of each amino acid (Table 2). These results demonstrated that acetate was converted to acetyl-CoA and incorporated into the TCA cycle under reductive operation. Moreover, a lower abundance of the labeled carbon in Ser and Asp supported the incomplete operation of the rTCA cycle (Figs 5 and 6). Accordingly, we conclude the occurrence of both the WL pathway and an incomplete rTCA cycle in *M. thermautotrophicus*, which is supported by the absence of genes for aconitase and isocitrate dehydrogenase [24, 25] and the previously reported ^14^C tracer-based metabolomics [22, 23].

**Table 2:** The isotopomer pattern of Ser, Asp, and Glu from the developed CE-MS/MS analysis.

	Isotopomer pattern		Natural abundance of isotopes	^13^CO_2_		^13^CO_2_ + acetate		CO_2_ + [1-^13^C_1_]acetate		CO_2_ + [2-^13^C_1_]acetate		Negative control	
	No. of ^13^C			Replicate 1	Replicate 2	Replicate 1	Replicate 2	Replicate 1	Replicate 2	Replicate 1	Replicate 2	Replicate 1	Replicate 2
Amino acid	Main chain	Side chain	Abundance (%)	Abundance (%)	Abundance (%)	Abundance (%)	Abundance (%)	Abundance (%)	Abundance (%)	Abundance (%)	Abundance (%)	Abundance (%)	Abundance (%)
^13^C_0_ Ser	0	0	96.74	84.99	92.89	80.51	86.59	95.73	96.23	95.19	94.04	96.89	96.86
^13^C_1_ Ser	1	0	1.08	4.06	2.69	6.29	3.54	0.96	1.24	1.24	1.14	1.07	0.97
	0	1	2.15	9.39	3.68	11.33	7.76	3.32	3.58	3.58	4.82	2.04	2.16
^13^C_2_ Ser	1	1	0.02	1.04	0.58	1.17	1.43	N.D.	N.D.	N.D.	N.D.	N.D.	N.D.
	0	2	0.01	0.52	0.16	0.70	0.68	N.D.	N.D.	N.D.	N.D.	N.D.	N.D.
^13^C_3_ Ser	1	2	0.00	N.D.	N.D.	0.12	0.04	N.D.	N.D.	N.D.	N.D.	N.D.	N.D.
^13^C_0_ Asp	0	0	95.67	78.93	89.01	71.48	80.21	95.26	94.66	93.77	92.55	95.88	96.28
^13^C_1_ Asp	1	0	1.06	4.51	2.43	5.66	3.91	0.88	1.30	1.08	1.34	1.31	0.96
	0	1	3.19	11.85	5.87	15.76	11.25	3.86	4.04	5.15	6.11	2.81	2.76
^13^C_2_ Asp	1	1	0.04	2.41	1.19	3.49	2.05	N.D.	N.D.	N.D.	N.D.	N.D.	N.D.
	0	2	0.04	1.90	1.23	2.85	2.21	N.D.	N.D.	N.D.	N.D.	N.D.	N.D.
^13^C_3_ Asp	1	2	0.00	0.40	N.D.	0.76	0.37	N.D.	N.D.	N.D.	N.D.	N.D.	N.D.
	0	3	0.00	N.D.	N.D.	N.D.	N.D.	N.D.	N.D.	N.D.	N.D.	N.D.	N.D.
^13^C_4_ Asp	1	3	0.00	N.D.	N.D.	N.D.	N.D.	N.D.	N.D.	N.D.	N.D.	N.D.	N.D.
^13^C_0_ Glu	0	0	94.62	71.81	84.11	65.74	73.99	93.05	93.52	92.35	91.52	94.67	94.54
^13^C_1_ Glu	1	0	1.05	4.14	4.19	6.11	5.27	1.83	1.09	1.50	1.37	1.26	1.20
	0	1	4.21	16.36	7.69	17.85	13.57	5.12	5.39	6.14	7.11	4.08	4.26
^13^C_2_ Glu	1	1	0.05	2.56	1.31	3.28	2.36	N.D.	N.D.	N.D.	N.D.	N.D.	N.D.
	0	2	0.07	3.95	1.83	5.26	3.71	N.D.	N.D.	N.D.	N.D.	N.D.	N.D.
^13^C_3_ Glu	1	2	0.00	0.77	0.56	1.18	0.75	N.D.	N.D.	N.D.	N.D.	N.D.	N.D.
	0	3	0.00	0.42	0.32	0.57	0.36	N.D.	N.D.	N.D.	N.D.	N.D.	N.D.
^13^C_4_ Glu	1	3	0.00	N.D.	N.D.	N.D.	N.D.	N.D.	N.D.	N.D.	N.D.	N.D.	N.D.
	0	4	0.00	N.D.	N.D.	N.D.	N.D.	N.D.	N.D.	N.D.	N.D.	N.D.	N.D.
^13^C_5_ Glu	1	4	0.00	N.D.	N.D.	N.D.	N.D.	N.D.	N.D.	N.D.	N.D.	N.D.	N.D.

N.D., not detected

The occurrence of both the WL pathway and an incomplete TCA cycle is consistent with a recent kinetic carbon network analysis that suggests acetyl-CoA influx from the WL pathway and/or other carbon metabolic pathways yielding acetyl-CoA conflicts with a complete rTCA cycle [31]. On the other hand, the acetate assimilation to acetyl-CoA in *M. thermautotrophicus* could be interpreted as an analog of the complete rTCA yielding acetyl-CoA. Thus, the capability of acetate assimilation likely contradicts the simulation. Recently, the acetate assimilation depending on available substrate concentration called acetate switch previously found in some bacteria and halophilic archaea [32-34] was also reported in a mesophilic and hydrogenotrophic methanogen *Methanococcus maripaludis* [35]*. M. maripaludis* likely also harbors an incomplete rTCA cycle in addition to the WL pathway. The biochemical mechanism behind the acetate assimilation in these hydrogenotrophic methanogens will provide novel insights into the operation of central carbon metabolism in methanogens and their metabolic evolution.

### Amino acid biosynthesis pathways of *M. thermautotrophicus*

The KEGG PATHWAY database is a visual collection of metabolic pathways and one of the most commonly used bioinformatic resources to identify enzymatic reactions and metabolic functions from genomic perspectives [27, 36]. The complete genome of *M. thermautotrophicus* in the KEGG PATHWAY database suggests that a number of reactions/enzymes in several amino acid biosynthesis pathways, including that for proline (Pro), cannot be clearly defined. The amino acid biosynthesis pathways obtained from the KEGG PATHWAY database were compared against the MetaCyc database, the most extensively curated collection of metabolic pathways containing 2749 pathways derived from more than 60 000 publications [28]. Then, GapMind [29] was used to curate the amino acid biosynthesis pathways and to identify candidate enzymes that were not present in the KEGG pathway (*M. thermautotrophicus*). GapMind is a web-based tool for annotating amino acid biosynthesis pathways in bacteria and archaea, where each curated pathway is supported by a confidence level [29]. However, three metabolic gaps in the amino acid biosynthesis pathways of *M. thermautotrophicus* still remained; genes for alanine (Ala) transaminase (EC 2.6.1.2), ornithine cyclodeaminase (EC 4.3.1.12), and homoserine kinase (EC 2.7.1.39), in the most probable Ala, Pro, and threonine (Thr) biosynthesis pathways, respectively.

To examine the probabilities of the amino acid biosynthesis pathways of *M. thermautotrophicus* in the KEGG PATHWAY database and MetaCyc, the mass isotopomer distributions of the detected 16 protein-derived amino acids in this methanogen were compared with the expected isotopomer structures predicted by GapMind (Supplementary Fig. S1 and Supplementary Table S1). In this analysis, glycine (Gly) was excluded because the *m/z* value of fragmented Gly was below the detection limit. In addition, a histidine (His) biosynthesis pathway could not be confirmed because the relative abundance of MS/MS spectra from fragmented His was not sufficient in cells labeled with [1-^13^C_1_] acetate or [2-^13^C_1_] acetate (Supplementary Table S1). All the amino acids detected in this study harbored one or more ^13^C (Supplementary Table S1). The labeling pattern of each amino acid was interpreted by the predicted amino acids biosynthesis pathways constructed from a combination of the public databases (KEGG PATHWAY and MetaCyc databases) and previously reported tracer-based metabolomics using radioisotope including Pro, Thr, and Ala biosynthesis pathways [37-39] (Supplementary Fig. S1). For example, the isotopomer patterns of Ser and aromatic amino acids suggested that pyruvate was their common precursor and supplied via gluconeogenesis (Supplementary Fig. S1 and Supplementary Tables S1 and S2).

Based on the isotopomer analysis, we also explored alternative enzymes to fill the metabolic gaps in the Ala, Pro, and Thr biosynthesis pathways again. The isotopomer pattern of Ala was similar to that of Ser, suggesting that Ala was synthesized from pyruvate by an alternative enzyme. Generally, this deamination is reversibly catalyzed by Ala dehydrogenase (EC 1.4.1.1) or Ala transaminase (EC 2.6.1.2). In the *M. thermautotrophicus* genome, a candidate gene (MTH_1495) showed significant amino acid sequence identity with a newly characterized archaeal Ala dehydrogenase in *Archaeoglobus fulgidus* [40, 41]. On the other hand, when we searched for Subgroup I transaminase, in which Ala transaminase was included [42], three genes (MTH_1894, MTH_1694, and MTH_52) were identified in their genome. Of these genes, MTH_1894 and MTH_1694 were annotated as Asp aminotransferase (EC 2.6.1.1), and MTH_52 was annotated as LL-diaminopimelate aminotransferase (EC 2.6.1.83). Transaminases often recognize a broad range of substrates, and predicting their specific substrate is challenging [43]. Thus, the archaeal Ala dehydrogenase and the three subgroup I transaminases in the *M. thermautotrophicus* genome could be candidates for an alternative enzyme for the reversible deamination between pyruvate and Ala. Similarly, in the case of Pro and Thr, the observed isotopomer structures were consistent with the most probable biosynthesis pathways, including previously unknown ornithine cyclodeaminase and homoserine kinase, respectively (Supplementary Fig. S1). Recently, it was reported that an archaeal ornithine cyclodeaminase (MMP_1218) in *M. maripaludis* produced Pro from ornithine [44]. In *M. thermautotrophicus*, MTH_867 showed significant amino acid sequence identity with MMP_1218 (58.75%), suggesting that archaeal ornithine cyclodeaminase encoded by MTH_867 filled the metabolic gap in the Pro biosynthesis pathway. However, no possible alternative enzymes were reported for the metabolic gap in the Thr biosynthesis pathway, and similar metabolic gaps are also observed in other microbes, such as *Methanocaldococcus jannaschii*, *Bacteroides thetaiotaomicron, Dinoroseobacter shibae*, and *Phaeobacter inhibens* [43].

The prediction of the amino acid biosynthesis pathways in public databases is beneficial to understand the metabolic and genetic capability of an organism, including its secondary metabolism. However, the limitations of the databases have been pointed out because they are based on biochemical, molecular biological, and genomic information from a limited number of model organisms, and thus, alternative biosynthesis pathways are often missing [43, 45]. The developed ^13^C tracer-based metabolomics using ZipChip CE system in combination with Orbitrap Fusion Tribrid mass spectrometer can help in predicting the most probable metabolic pathway and provide clues to reveal the presence of unknown alternative enzymes or unknown amino acid biosynthesis pathways that are not recognized in the public database. Due to the high sensitivity and accuracy of the CE-MS system, the developed tracer-based metabolomic method can identify metabolic pathways and fluxes related to amino acid biosynthesis in any kind of microbe. In addition, the method is applicable to cells under biomass-restricted conditions, even in model organisms, such as cells in early growth phases or cells under some kind of growth suppression. The simple method is a promising analytical tool to construct more reliable databases of microbial metabolic pathways.

## Conflicts of interest

The authors declare no competing interests.

## Funding

This work was supported by the Grant-in-Aid for Scientific Research on Innovative Areas “Post-Koch Ecology” (19H05684) and (A) (19H00988) from the Ministry of Education, Culture, Sports, Science and Technology (MEXT). The Green Innovation Fund Project, JPNP22010, commissioned by the New Energy and Industrial Technology Development Organization (NEDO).

## Data availability

The most relevant data are included in this published article and its supplementary information files. Raw data generated during the current study are not publicly available, but are available from the corresponding author upon reasonable request.

## Supplementary Material

Fukuyama_etal_ISMECOMMS-23-00275A_revised_SuppleFigure_ycad006

Fukuyama_etal_ISMECOMMS-23-00275A_revised_SuppleTable_ycad006
