# Supplementary material for: Development of a rapid and highly accurate method for 13C tracer-based metabolomics and its application on a hydrogenotrophic methanogen
Source: ISME Commun. 2024 Jan 10;4(1):ycad006. doi: 10.1093/ismeco/ycad006 (PMC10809761; doi:10.1093/ismeco/ycad006)
Supplement: Fukuyama_etal_ISMECOMMS-23-00275A_revised_SuppleFigure_ycad006 [file fukuyama_etal_ismecomms-23-00275a_revised_supplefigure_ycad006.pdf]

Glycolysis / Gluconeogenesis

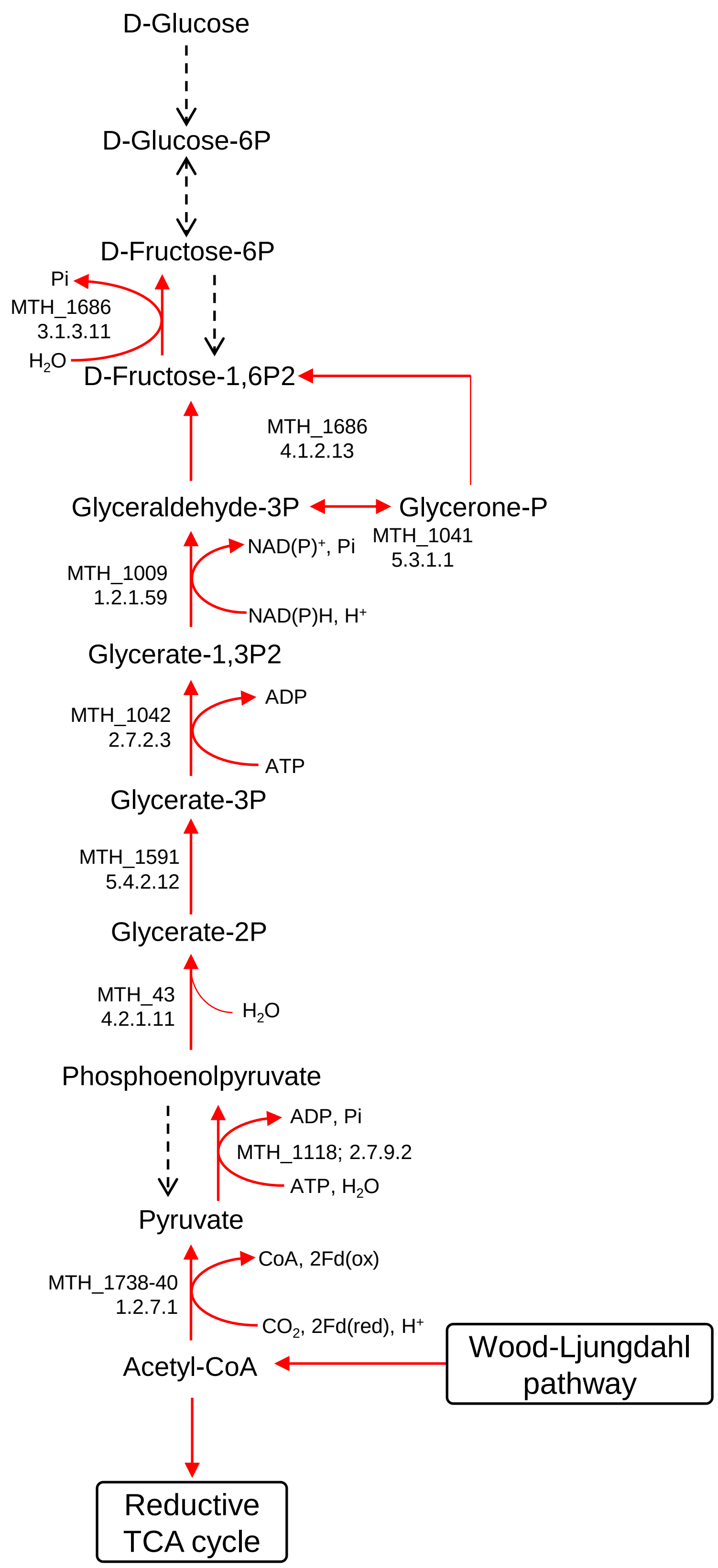

Wood-Ljungdahl pathway

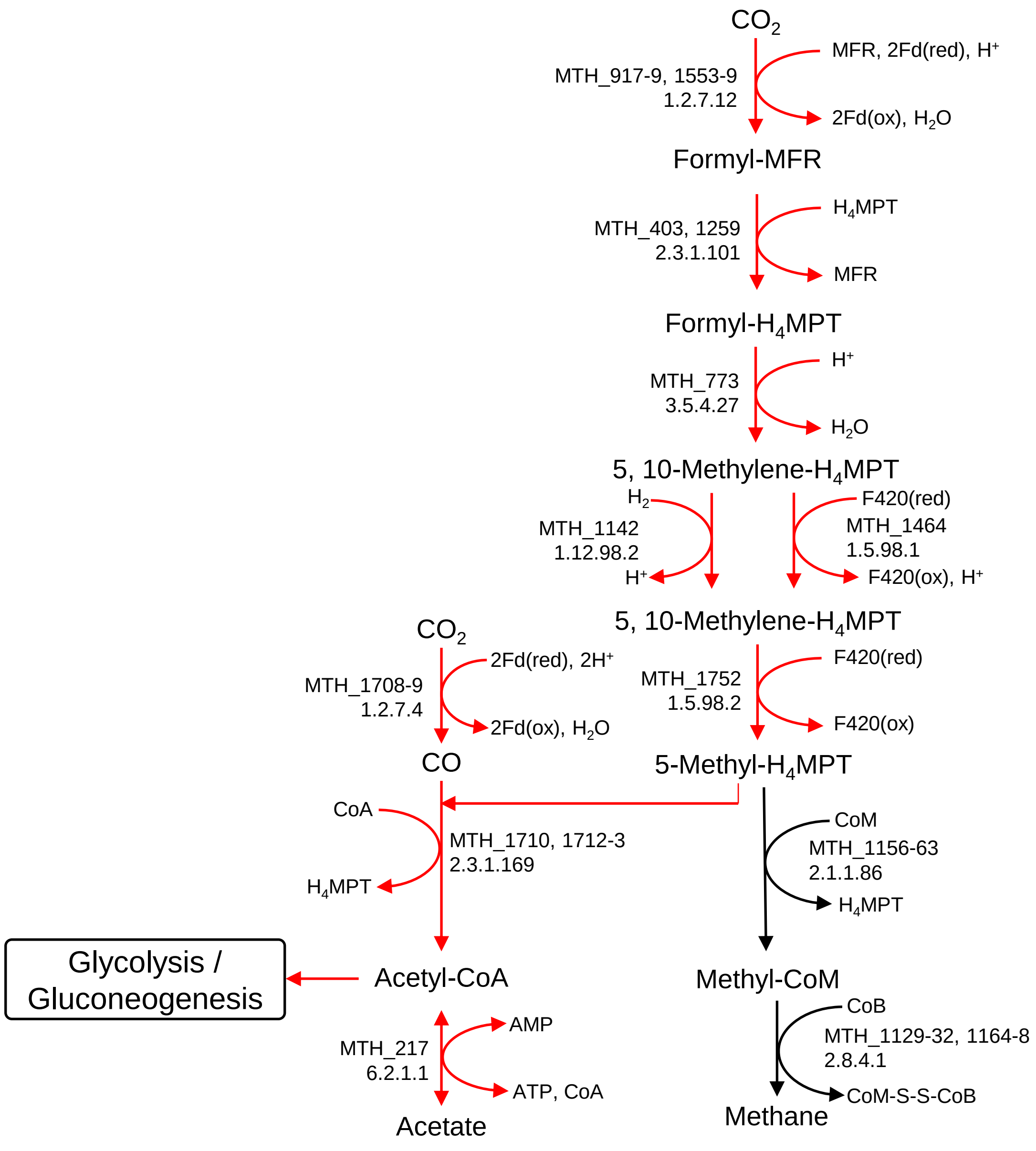

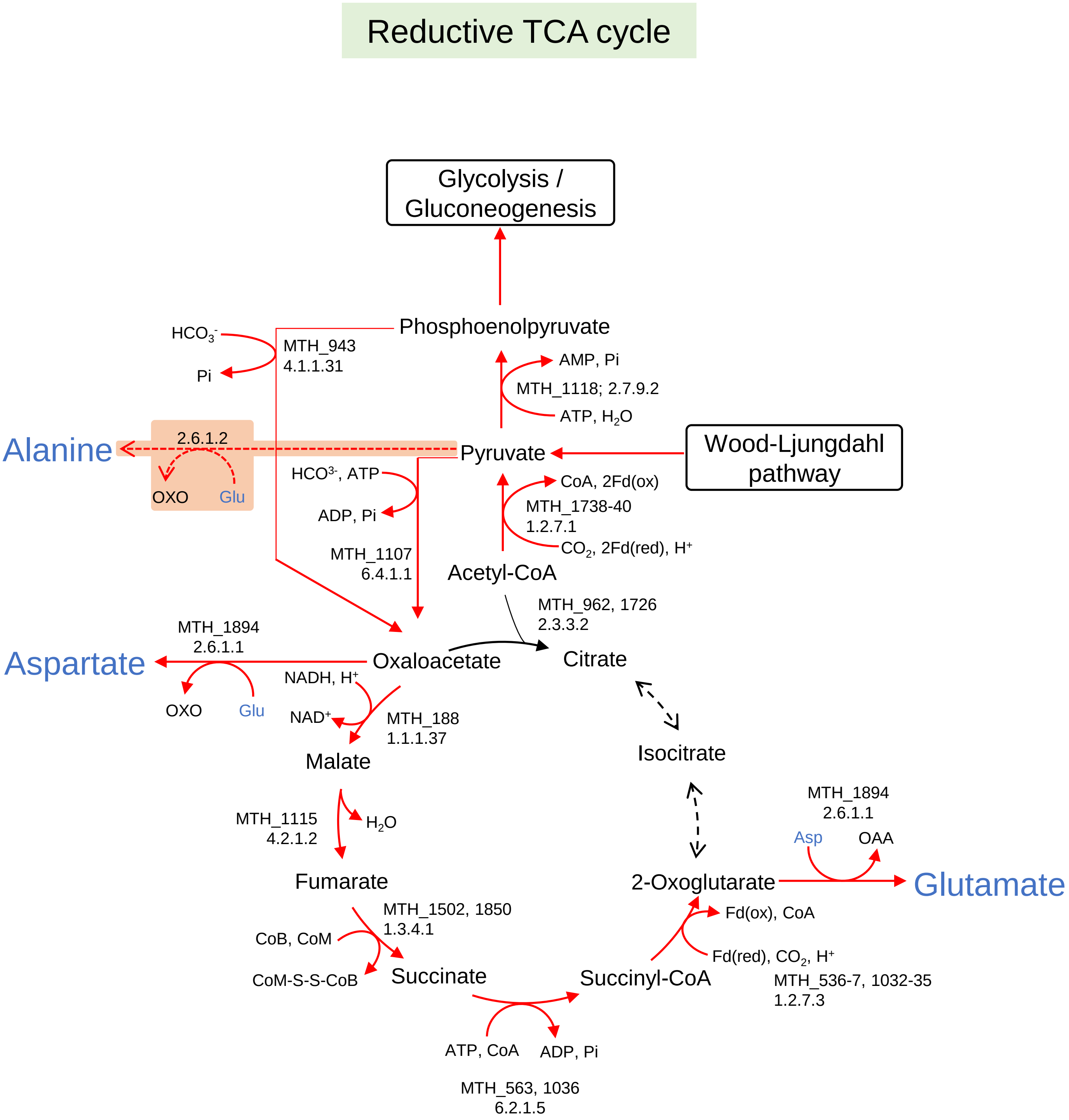
Reductive TCA cycle
Glycolysis / Gluconeogenesis
Phosphoenolpyruvate
HCO3-
Pi
MTH_943
4.1.1.31
AMP, Pi
MTH_1118; 2.7.9.2
ATP, H2O
Alanine
2.6.1.2
OXO
Glu
Pyruvate
Wood-Ljungdahl pathway
HCO3-, ATP
ADP, Pi
MTH_1107
6.4.1.1
CoA, 2Fd(ox)
MTH_1738-40
1.2.7.1
CO2, 2Fd(red), H+
Acetyl-CoA
MTH_962, 1726
2.3.3.2
MTH_1894
2.6.1.1
Aspartate
Oxaloacetate
Citrate
OXO
Glu
NADH, H+
NAD+
MTH_188
1.1.1.37
Malate
Isocitrate
MTH_1115
4.2.1.2
H2O
MTH_1894
2.6.1.1
Asp
OAA
Fumarate
2-Oxoglutarate
Glutamate
MTH_1502, 1850
1.3.4.1
Fd(ox), CoA
CoB, CoM
CoM-S-S-CoB
Succinate
Succinyl-CoA
Fd(red), CO2, H+
MTH_536-7, 1032-35
1.2.7.3
ATP, CoA
ADP, Pi
MTH_563, 1036
6.2.1.5

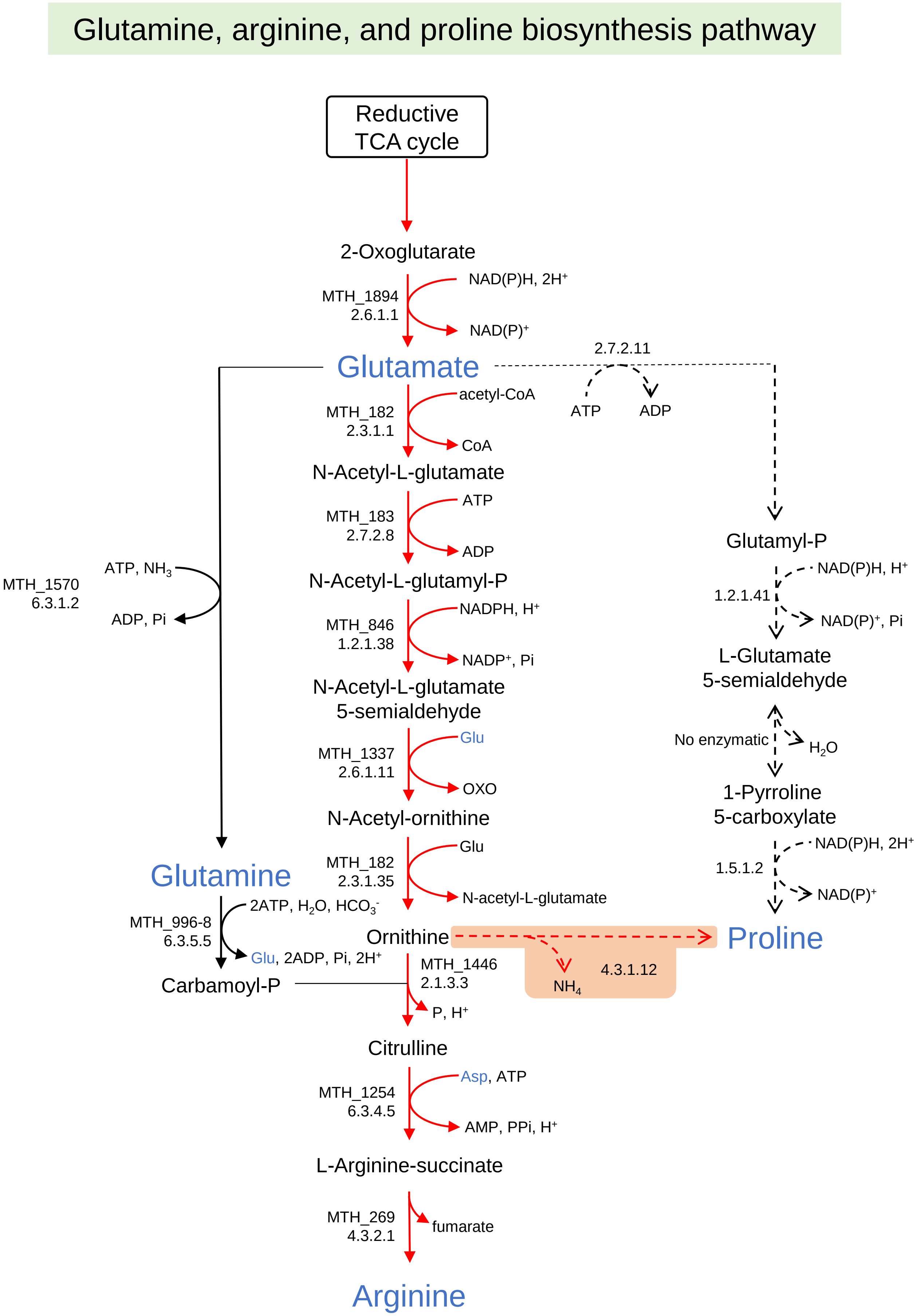

Glutamine, arginine, and proline biosynthesis pathway
Reductive TCA cycle
2-Oxoglutarate
MTH_1894
2.6.1.1
NAD(P)H, 2H+
NAD(P)+
Glutamate
2.7.2.11
ATP
ADP
MTH_182
2.3.1.1
acetyl-CoA
CoA
N-Acetyl-L-glutamate
MTH_183
2.7.2.8
ATP
ADP
N-Acetyl-L-glutamyl-P
MTH_846
1.2.1.38
NADPH, H+
NADP+, Pi
N-Acetyl-L-glutamate 5-semialdehyde
MTH_1337
2.6.1.11
Glu
OXO
N-Acetyl-ornithine
MTH_182
2.3.1.35
Glu
N-acetyl-L-glutamate
Ornithine
MTH_1446
2.1.3.3
P, H+
Citrulline
MTH_1254
6.3.4.5
Asp, ATP
AMP, PPi, H+
L-Arginine-succinate
MTH_269
4.3.2.1
fumarate
Arginine
MTH_1570
6.3.1.2
ATP, NH3
ADP, Pi
Glutamine
MTH_996-8
6.3.5.5
2ATP, H2O, HCO3-
Glu, 2ADP, Pi, 2H+
Carbamoyl-P
Glutamyl-P
1.2.1.41
NAD(P)H, H+
NAD(P)+, Pi
L-Glutamate 5-semialdehyde
No enzymatic
H2O
1-Pyrroline 5-carboxylate
1.5.1.2
NAD(P)H, 2H+
NAD(P)+
Proline
4.3.1.12
NH4

Glycine, serine, and cysteine biosynthesis pathway

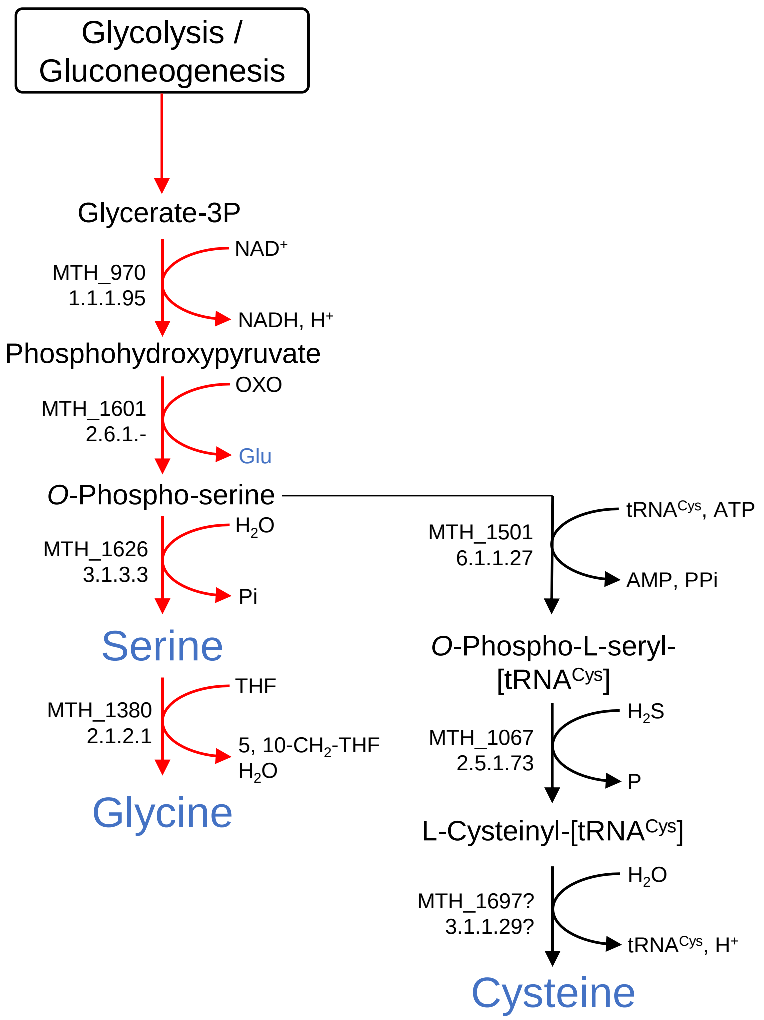

Lysine, threonine, methionine, and asparagine biosynthesis pathway

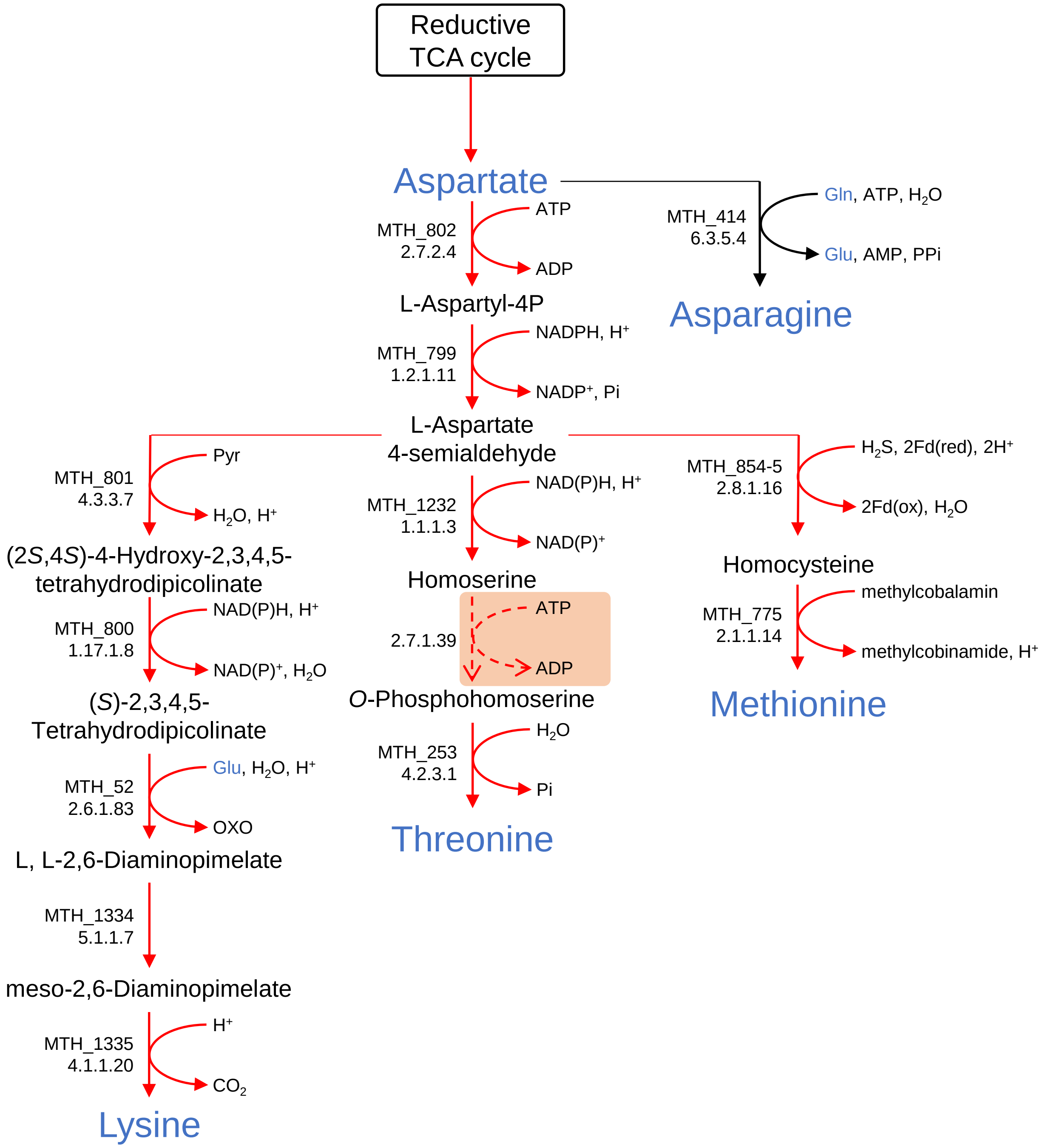

Histidine biosynthesis pathway

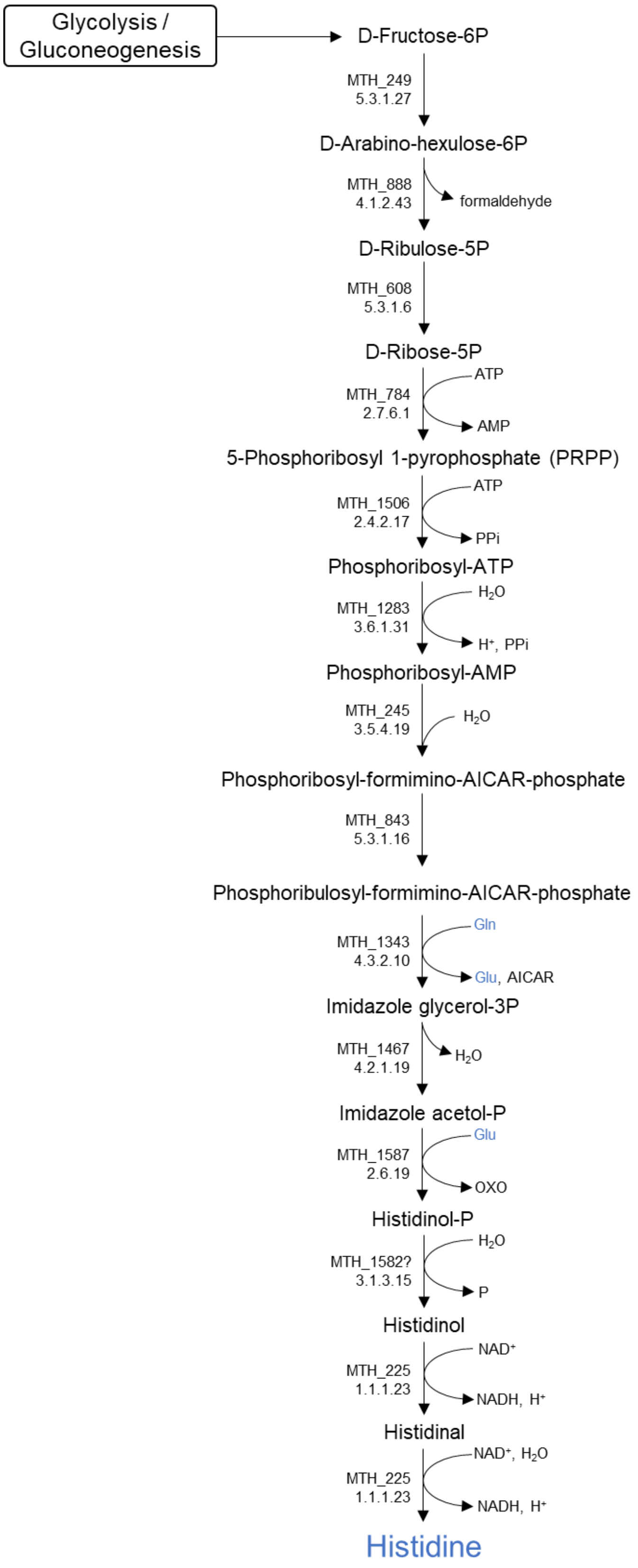

Tryptophan, phenylalanine, and tyrosine biosynthesis pathway

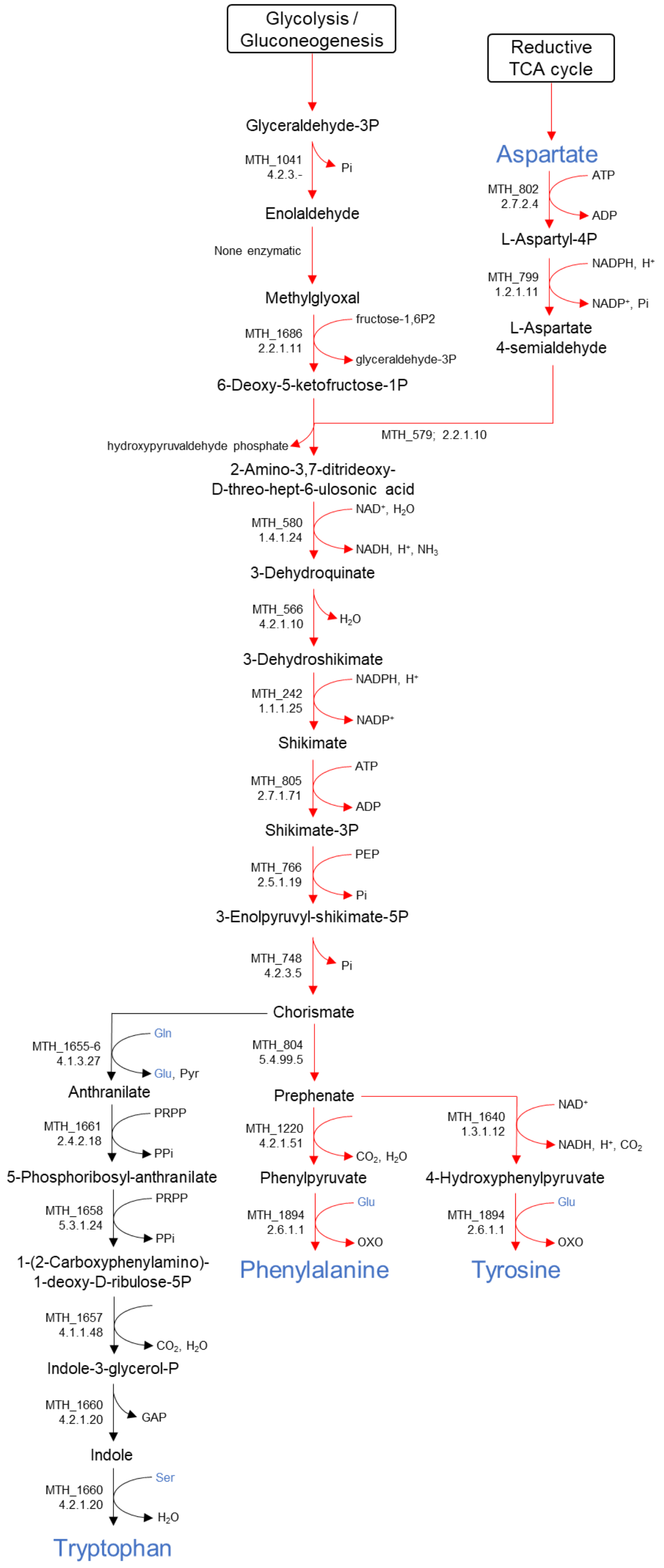

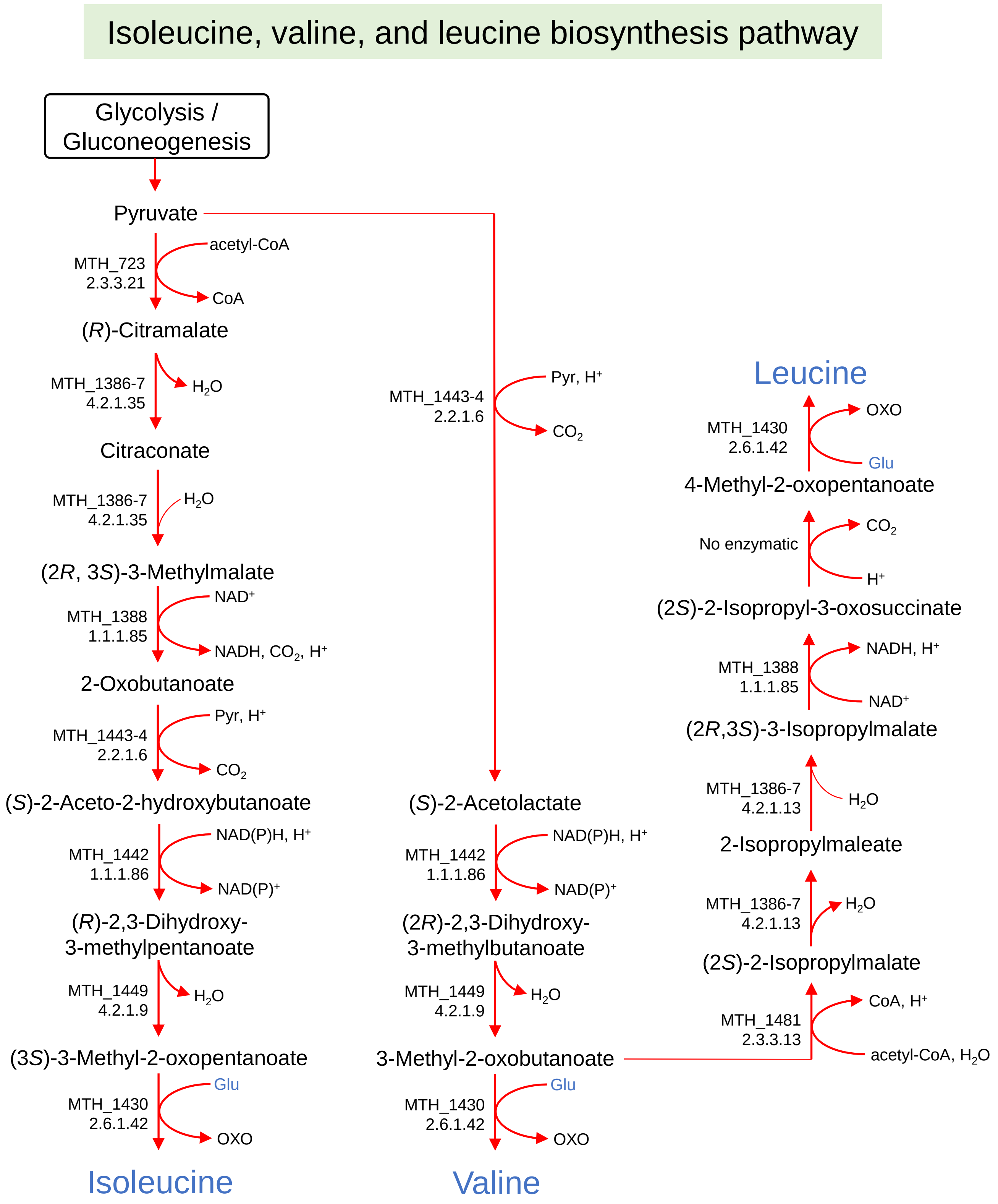

Fig. S1 Central carbon metabolism and amino acid biosynthetic pathway of *M. thermautotrophicus* under autotrophic growth condition reconstructed from the genome information and isotopomer analysis using the developed CE-MS and CE-MS/MS techniques

Compounds colored in blue indicate proteinogenic amino acids. Red lines indicate active pathways under autotrophic growth condition confirmed by isotopomer analysis. Dot lines indicate there are no corresponding enzymes found in the genome. The pathways predicted to be present using the tracer-based metabolomics are highlighted in orange. Among 20 proteinogenic amino acids, biosynthetic pathways of asparagine, glutamine, tryptophan, cysteine, and histidine are not conformed in this study.
